# Supplementary figures and images for: In Vitro Anticancer Activity and Oxidative Stress Biomarkers Status Determined by Usnea barbata (L.) F.H. Wigg. Dry Extracts
Source: Antioxidants (Basel). 2021 Jul 20;10(7):1141. doi: 10.3390/antiox10071141 (PMC8301184; doi:10.3390/antiox10071141)

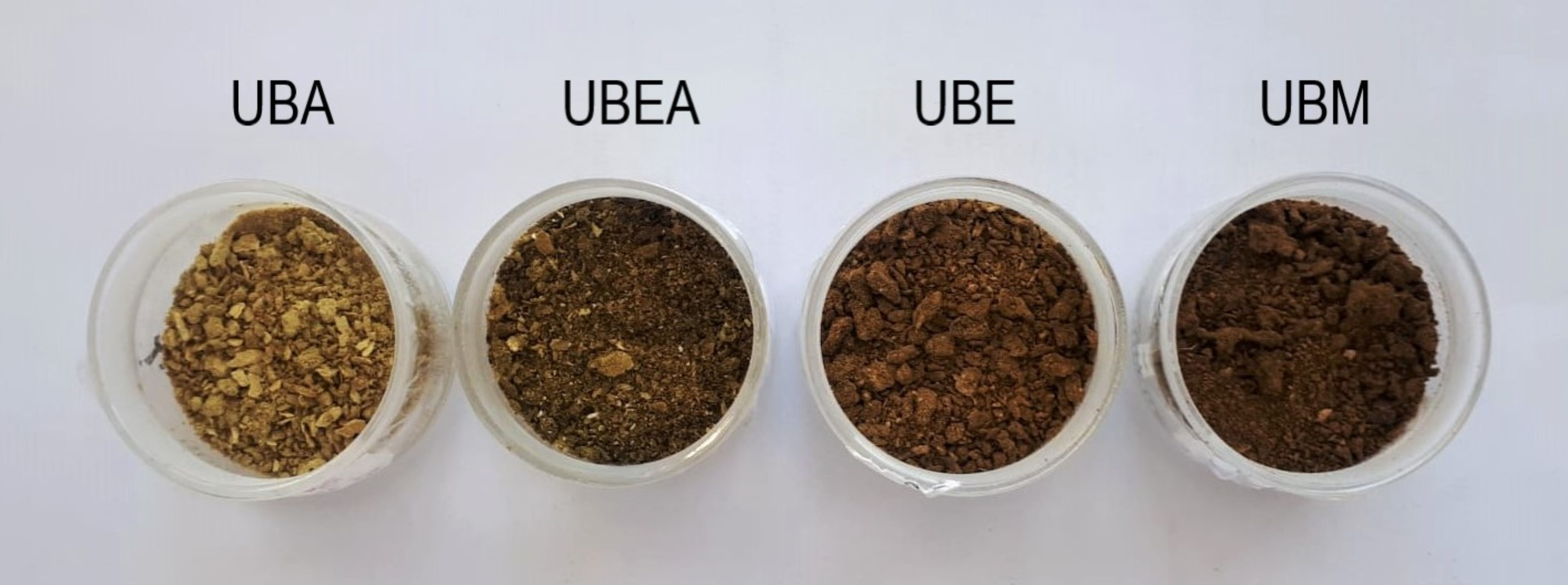

Supplement: Supplementary file 1 [file antioxidants-10-01141-s001.zip › Figure S1.tiff]

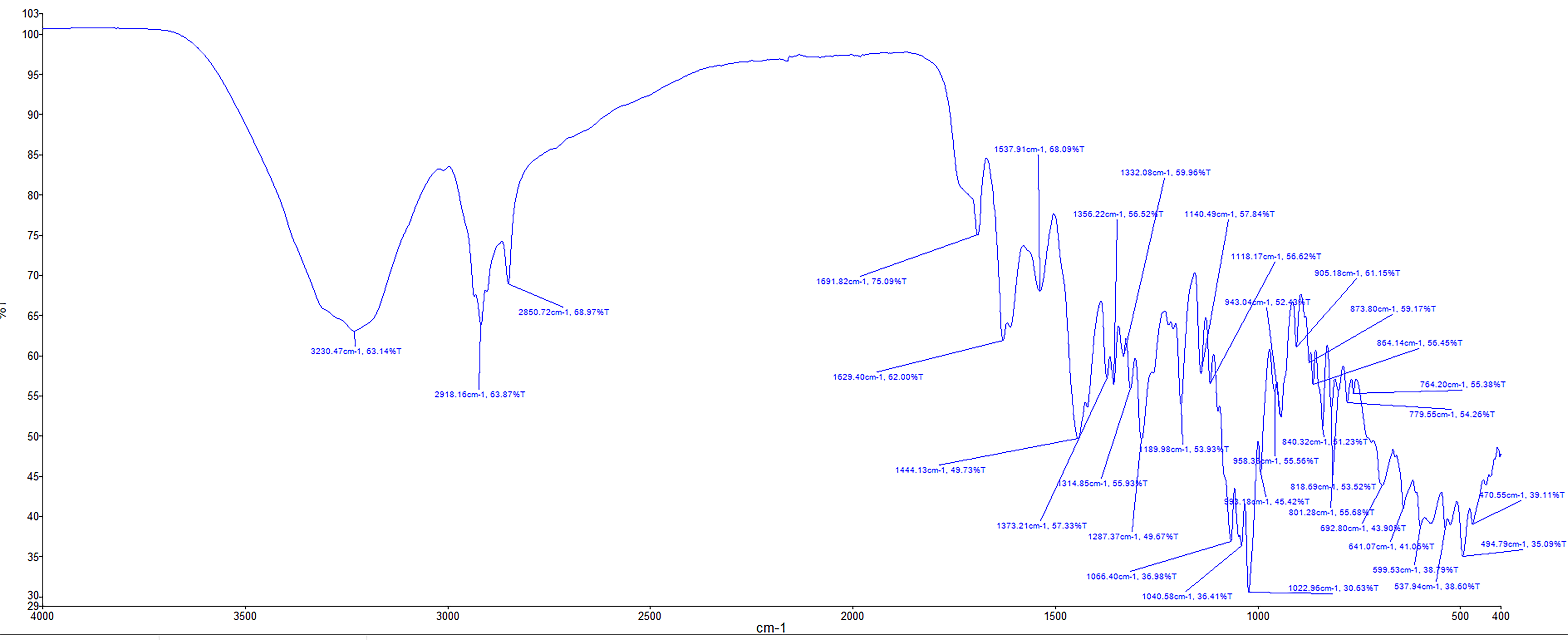

Supplement: Supplementary file 1 [file antioxidants-10-01141-s001.zip › Figure S2.tif]

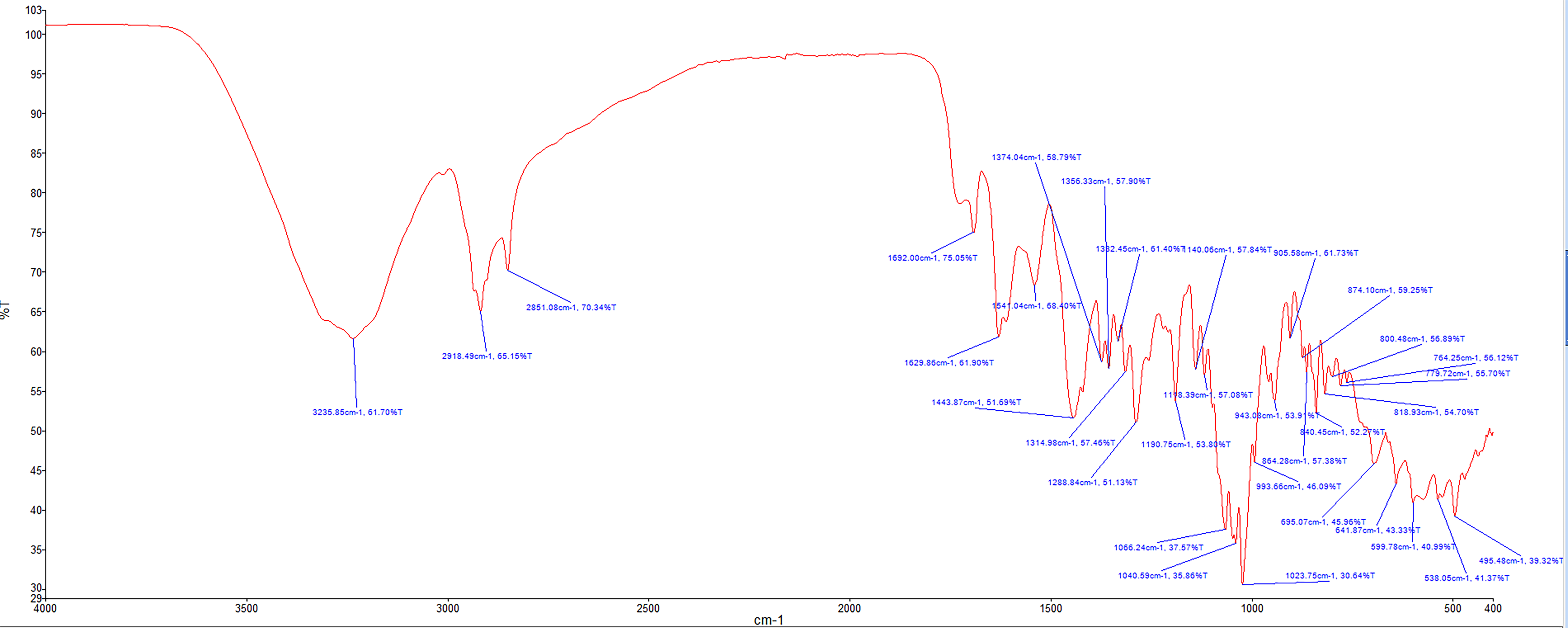

Supplement: Supplementary file 1 [file antioxidants-10-01141-s001.zip › Figure S3.tif]

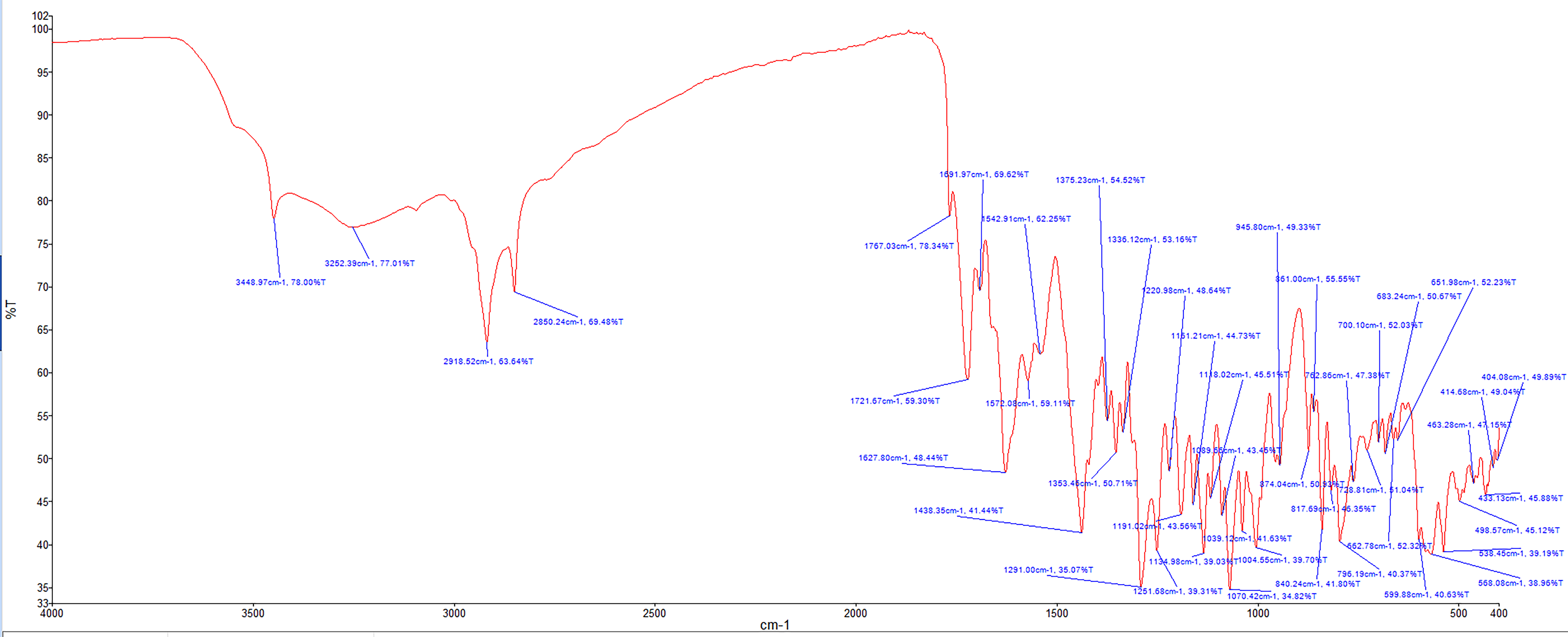

Supplement: Supplementary file 1 [file antioxidants-10-01141-s001.zip › Figure S4.tif]

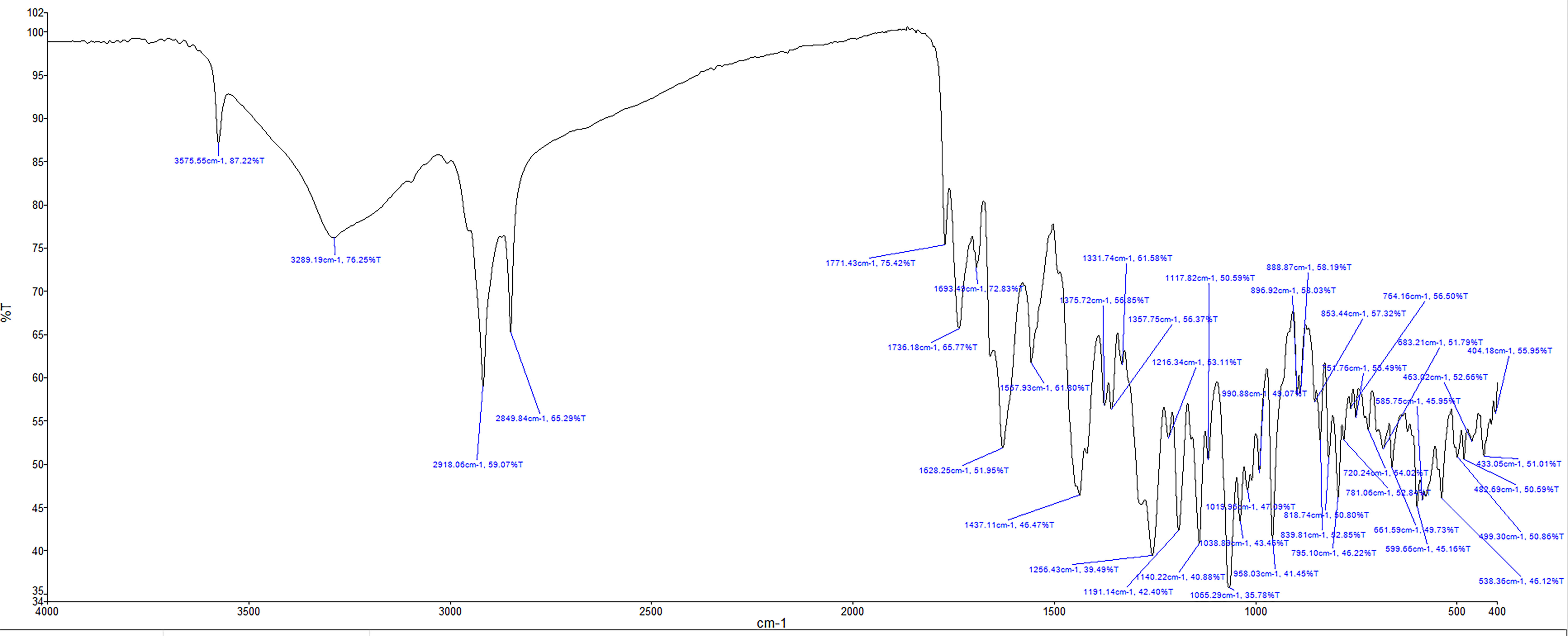

Supplement: Supplementary file 1 [file antioxidants-10-01141-s001.zip › Figure S5.tif]

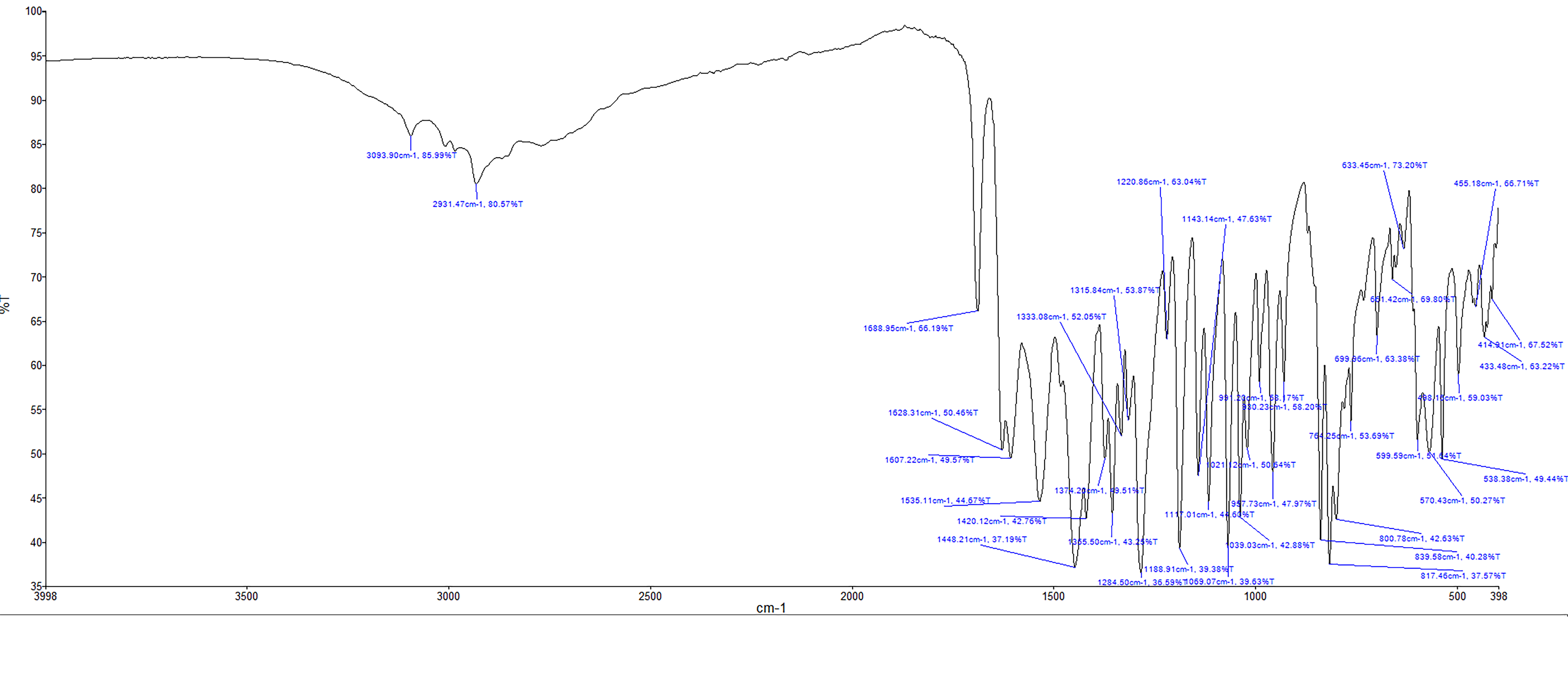

Supplement: Supplementary file 1 [file antioxidants-10-01141-s001.zip › Figure S6.tif]

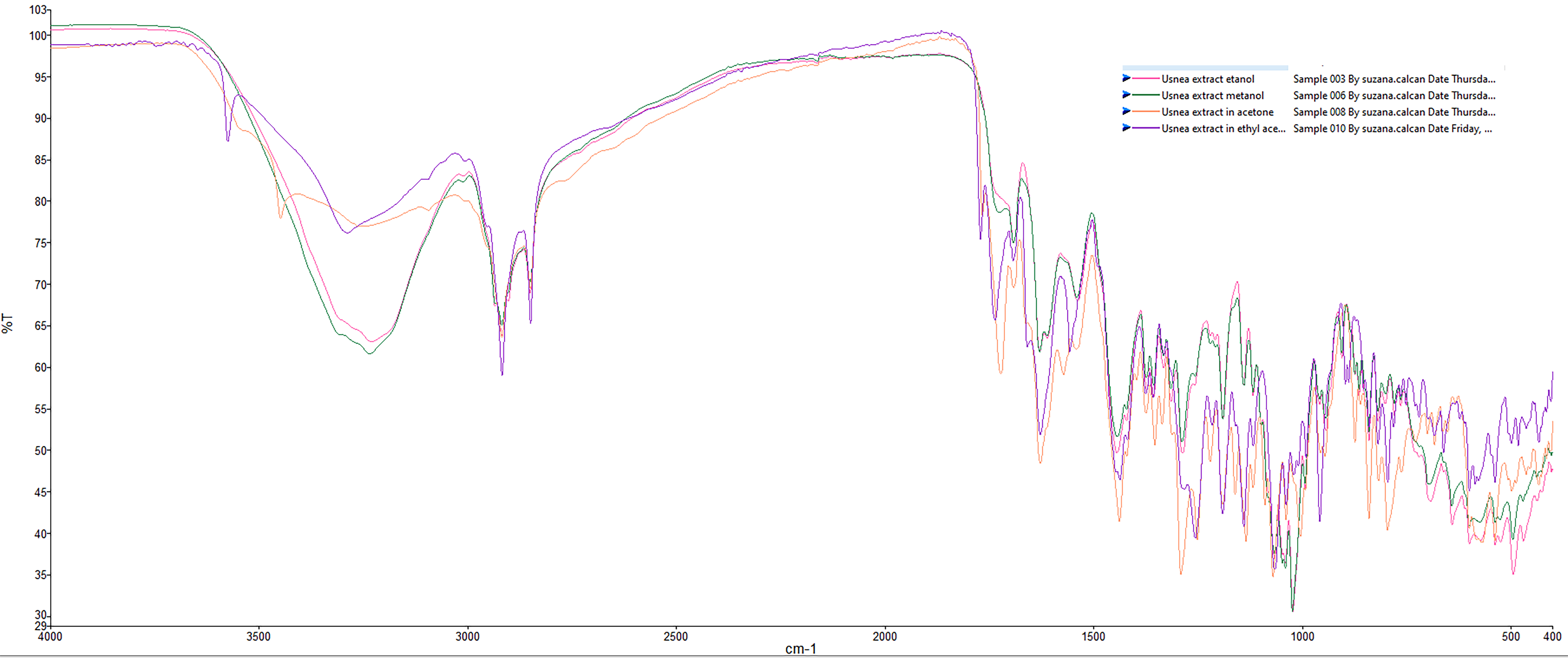

Supplement: Supplementary file 1 [file antioxidants-10-01141-s001.zip › Figure S7.tif]
